# Impact of Pharmacist Interventions in a Portuguese Hospital: A Study Using the CLEO Multidimensional Tool

**DOI:** 10.3390/pharmacy13050143

**Published:** 2025-10-05

**Authors:** Sofia Silva, Mafalda Jesus, Sandra Faria, Sara Machado, Manuel Morgado

**Affiliations:** 1 Pharmaceutical Services of Local Healthcare Unit of Matosinhos, 4464-513 Senhora da Hora, Portugal; sofia.pinto@ulsm.min-saude.pt (S.S.); sandra.silvafaria@ulsm.min-saude.pt (S.F.); 2 RISE-Health, Department of Medical Sciences, Faculty of Health Sciences, University of Beira Interior, Av. Infante D. Henrique, 6200-506 Covilhã, Portugal; mafalda.jesus@ubi.pt; 3 Hospital José Joaquim Fernandes, Unidade Local de Saúde do Baixo Alentejo, 7800-491 Beja, Portugal; s.machado@edu.ulisboa.pt; 4 iMed.ULisboa and Departamento de Farmácia, Farmacologia e Tecnologias de Saúde, Faculdade de Farmácia, Universidade de Lisboa, 1649-003 Lisbon, Portugal; 5 Faculty of Health Sciences, University of Beira Interior (FCS-UBI), 6200-506 Covilhã, Portugal; 6 Pharmaceutical Services of Local Health Unit of Cova da Beira (ULS Cova da Beira), 6200-251 Covilhã, Portugal

**Keywords:** pharmacist interventions, oncology pharmacy, drug-related problems, CLEO tool, clinical and economic impact, patient safety

## Abstract

(1) Background: Pharmacist interventions are key to optimizing medication therapy and improving patient outcomes. The CLEO multidimensional tool assesses the clinical, economic, and organizational impact of these interventions, though its use in Portuguese hospital settings is limited. This study explored the predicted impact of pharmacist interventions in the Oncology Department of a Portuguese hospital, using CLEO to quantify their potential contribution to patient care and healthcare system efficiency;(2) Methods: A retrospective observational study was conducted at the hospital’s Oncology Outpatient Pharmacy between April and December 2024. Data from 144 pharmacist interventions were analyzed, focusing on drug-related problems, corrective actions, and CLEO scores. Descriptive statistics were used for data analysis; (3) Results: The most frequent drug-related problems were incorrect administration frequency (57.6%), drug interactions (22.2%), and incorrect dosing (10.4%). Nearly half of the interventions (47.2%) resulted in prescription corrections. CLEO analysis demonstrated a predicted positive clinical impact (80% of interventions scored 1C–3C), potential economic benefits (40.3% scored 1E), and organizational improvements (79.9% scored 1O), especially in lung, breast, and colorectal cancer treatments; (4) Conclusions: Pharmacist interventions were predicted to be associated with improvements in clinical, economic, and organizational outcomes in oncology care. These findings suggest that systematic documentation and evaluation of interventions using CLEO may enhance patient safety and healthcare efficiency, although further multicenter and prospective studies are needed to confirm these observations.

## 1. Introduction

Pharmacist interventions (PIs) play a critical role in optimizing pharmacotherapy and improving patient outcomes across diverse healthcare settings. Clinical pharmacists are increasingly integrated into multidisciplinary care teams, particularly in hospital environments, where they routinely participate in ward rounds, review medication regimens, and recommend evidence-based therapeutic adjustments. These interventions are typically initiated upon identifying drug-related problems (DRPs) or medication discrepancies and often involve direct communication with prescribers to recommend changes in drug selection, dose, route of administration, or treatment duration. Studies have demonstrated that such interventions are a frequent and significant component of clinical practice [1,2].

Defined as “any professional activity by the pharmacist directed towards improving the quality use of medicines and resulting in a recommendation for a change in the patient’s medication therapy, means of administration, or medication-taking behavior” [3], PIs have been consistently associated with reductions in medication errors, improved disease management, and enhanced safety profiles and lower healthcare costs [4,5,6]. Their value is further supported by evidence linking clinical pharmacy services to reduced hospital mortality rates and improved therapeutic efficiency [7,8].

The systematic documentation of pharmacist clinical activities, including PIs, is essential not only for ensuring continuity of care but also for supporting multidisciplinary collaboration, quantifying workload, and evaluating the quality of pharmaceutical services [9,10,11]. Additionally, structured documentation contributes to the identification of DRPs, informs institutional guidelines, supports training and professional development, and provides essential data for healthcare planning [12]. Nevertheless, documentation practices vary considerably between institutions and countries, and challenges persist in the consistent and standardized assessment of the impact of pharmacists’ contributions [13].

To support a more structured approach, the CLEO tool was developed and validated by Vo et al. [5] and endorsed by the French Society of Clinical Pharmacy. This tool enables a structured, multidimensional assessment of each PI across three independent domains: (a) Clinical impact (e.g., prevention of adverse events, improvement in therapeutic efficacy); (b) Economic impact (e.g., cost savings, avoidance of unnecessary treatments); (c) Organizational impact (e.g., workflow optimization, avoidance of care delays). CLEO has demonstrated good feasibility and reliability in different healthcare contexts and has been integrated into national-level initiatives such as the French National Observatory—Act-IP^©^ [14]. It has also been translated and validated for use in other healthcare systems, including Switzerland and Vietnam [7,15], which further supports its potential for broader applicability. Recent studies have demonstrated CLEO’s applicability across several healthcare settings [16,17,18].

However, further studies are needed to further investigate CLEO’s use in different clinical specialties and healthcare systems, particularly in southern European contexts. This study aims to assess the clinical, economic, and organizational impact of PIs performed in the Oncology Department of a secondary hospital in the North of Portugal. By systematically evaluating interventions using the CLEO tool, this research seeks to generate evidence on the value of pharmacists’ contributions to patient care and to support efforts toward more structured documentation of clinical pharmacy activities.

This study aims to assess the clinical, economic, and organizational impact of PIs performed in the Oncology Department of a secondary hospital in the North of Portugal. By systematically evaluating PIs using the CLEO tool, this research seeks to generate evidence on the contribution of pharmacists in PIs to patient care and to promote structured documentation practices in clinical pharmacy.

## 2. Materials and Methods

### 2.1. CLEO Tool

The CLEO tool was developed through a review of previous models and tools for evaluating PIs, as well as by incorporating the expertise of clinical pharmacists [5,19].

The tool was developed under the assumption that individual pharmaceutical interventions mainly influence care processes and outcomes, with limited direct impact on healthcare system structures. CLEO allows for a standardized assessment of the potential impact of pharmaceutical interventions in each of these dimensions [5].

Thus, this tool assesses three dimensions: Clinical, Economic, and Organizational [5].

Clinical dimension (Score C): evaluates, from the viewpoint of the patient, the predicted effects of a PI on the patient’s health. It consists of a six-point scale that goes from −1 (harmful) to 4 (prevention of a fatal consequence). This structure was adapted from Hatoum et al.’s classification and the NCC MERP index, which are widely recognized for assessing clinical relevance and medication error severity [5].

Organizational dimension (Score O): analyzes the potential impact of the PIs on healthcare procedures from the perspective of the provider. It considers elements like enhanced workplace safety, decreased professional burden, and optimization of time and resources. It employs a three-point rating system (−1 to 1) where −1 means a negative and 1 a positive impact [5].

Economic dimension (Score E): focuses on the hospital’s perspective regarding the PI’s predicted impact on direct treatment costs. It uses a three-point scale: −1 (increase in cost), 0 (no change), and 1 (cost saving). Cost avoidance is intentionally excluded, as it is partially reflected in the clinical dimension; this scale is designed to capture only direct cost reductions. The structure is based on Briceland et al.’s economic classification [5].

### 2.2. Study Design

This retrospective, single-center, descriptive observational study was conducted at the Oncology Outpatient Pharmacy of the Day Hospital of the Matosinhos Local Health Unit, Portugal. It included PIs performed between April and December 2024.

The inclusion criteria were: (1) Patients aged 18 years or older; (2) Patients receiving treatment at the Oncology Outpatient Pharmacy; (3) Patients with solid tumors, hematological malignancies, or selected non-malignant hematological conditions in whom pharmaceutical interventions have been carried out.

Patients who did not meet these criteria were excluded. Eligibility was determined by reviewing the PI’s records from the study period.

All eligible patients who met the inclusion criteria and received medication interventions during the study period were included; therefore, no sampling method was applied. As a result, the sample size was not determined beforehand. During the study period, PIs were recorded in 128 patients. Of these, three patients were excluded because, although they received medications dispensed at the Oncology Outpatient Pharmacy, the treatments were prescribed for non-oncological conditions and therefore did not meet the inclusion criteria. Consequently, the final study population comprised 125 oncology patients, in whom a total of 144 PIs were documented. This approach ensured that the dataset accurately reflected the real-world clinical activity of the Oncology Outpatient Pharmacy, without sampling or selection bias.

### 2.3. Registration Routine

PIs were documented using an Excel-based form accessible via the Teams platform, linked to the institutional emails of all hospital pharmacists working at the Oncology Outpatient Pharmacy. This form was developed based on the 2nd Granada Consensus on DRPs [20,21,22]. This Consensus establishes six main criteria of pharmacotherapy failure ((1) untreated health problem, (2) effect of unnecessary drug treatment, (3) non-quantitative ineffectiveness, (4) quantitative ineffectiveness, (5) non-quantitative safety problem, and (6) quantitative safety problem. Based on these six criteria, 21 categories of DRPs were created in our registration form (which are detailed in Supplementary Table S1).

### 2.4. Data Collection and Classification

Data were extracted from the Excel form for the defined analysis period (April–December 2024), and only PIs meeting the predefined inclusion criteria were selected. Two experienced hospital clinical pharmacists participated in data collection and applied the CLEO multidimensional tool to the PIs, and any disagreements were resolved by a third pharmacist. As the reviewers were pharmacists from the same service, the assessment may have been subject to professional bias; no independent evaluation by physicians or nurses was performed. Authorization for the use of the CLEO tool was obtained from its authors.

Each recorded intervention was assessed retrospectively using the CLEO tool. Documentation in the electronic Excel form served as the basis for assessment. In cases where classification was uncertain, the pharmacy team reached a consensus through discussion. Each intervention received an individual score for the three CLEO dimensions (Clinical, Economic, and Organizational), enabling a multidimensional evaluation of each intervention’s predicted impact.

### 2.5. Ethical Considerations

The Matosinhos Local Health Unit’s Ethics Committee gave its approval to this study (reference number 0022/CES/JAS 14 March 2025). Anonymized data collected during routine clinical practice were retrospectively analyzed. Patient confidentiality was ensured in accordance with the General Data Protection Regulation (GDPR). The original English version of the CLEO tool, which is freely accessible in the scientific literature, was used in accordance with its disclosed methodology. 

All pharmacists had adequate English skills to use the tool correctly, and therefore, no official translation or cultural adaptation was performed.

### 2.6. Data Analysis

An internal database was created to consolidate study variables. Descriptive statistical analyses were performed using Microsoft Excel to quantify and summarize the impact of PIs. The analysis was based on absolute and relative frequencies to describe the distribution of variables.

## 3. Results

### 3.1. Patient Characteristics

A total of 144 PIs were recorded over a 9 month period. These interventions may have involved fewer unique patients, as some individuals could have received more than one PI. 

Most of these PIs were given to female patients (86; 59.7%) in the age groups 18–64 (47; 32.6%) and 65–85 (93; 64.6%) years. More precisely, the PIs were predominantly concentrated among patients aged over 40 years, with a notable prevalence in the 65–85 age group. Breast cancer (29; 20.1%) and lung cancer (29; 20.1%) were the pathologies that received the most PIs, followed by colorectal cancer (16; 11.1%), prostate cancer (15; 10.4%) and multiple myeloma (13; 9.0%). These pathologies are typically associated with intensive pharmacological treatment, which highlights the relevance of pharmacist involvement. Table 1 presents the baseline demographic and clinical characteristics of the patients included in the study who received PIs.

### 3.2. Active Substances Involved in Interventions

Serotonin (5-HT3) antagonists, especially ondansetron (28; 19.4%), were the agents that received the highest number of PIs. Immunostimulants, such as filgrastim (20; 13.9%), and dexamethasone (18; 12.5%), also received a similar number of interventions. Table 2 shows the active substances involved in PIs.

### 3.3. Drug-Related Problems and Actions Taken

In the present study, only eight out of the twenty-one predefined categories were identified: (1) Incorrect frequency or time of administration; (2) Drug–drug interaction (manifest or potential); (3) Incorrect dose (too high or too low); (4) Medication omission; (5) Inconsistent information; (6) Duplication of therapy; (7) Medication not indicated or appropriate for the diagnosis; (8) Other reasons.

**Figure 1:**
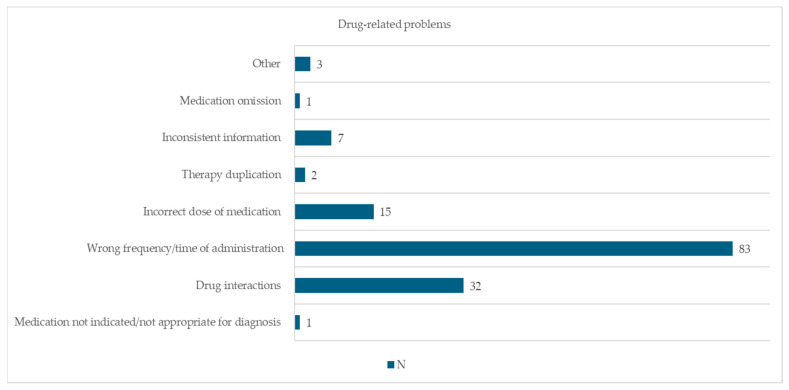
Frequencies of DRPs identified by clinical pharmacists.

The most common DRP was wrong frequency/time of administration (83; 57.6%), followed by drug interactions (32; 22.2%) and wrong dose (15; 10.4%). No life-threatening DRPs were identified. “Other reasons” refers to all DRPs that could not be directly classified into one of the 20 established DRPs. Figure 1 shows the clinical pharmacist’s assessment of the different DRPs identified. A full description of the DRP categories considered for the study can be found in Supplementary Table S1.

Most of the PIs involved a correction of the medical prescription (68; 47.2%) as an action taken to address the DRPs described above, followed by maintenance of the medical prescription (43; 29.9%). Not applicable (33; 22.9%) refers to all PIs that did not require medical consultation. A quantitative schematic representation of the actions taken during PIs can be found in Supplementary Table S2.

### 3.4. CLEO Analysis

**Figure 2:**
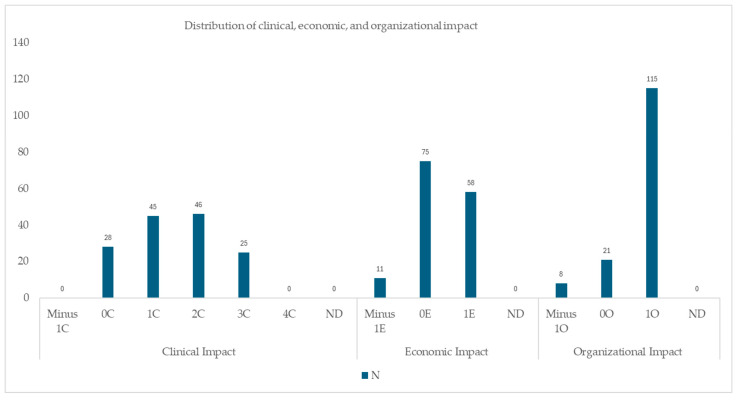
CLEO impact assessment according to PIs conducted in the study.

The clinical, economic and organizational impact was assessed by clinical pharmacists. The results suggest that, overall, PIs had a positive impact on the three dimensions of the tool. In terms of clinical impact, 1C, 2C and 3C scores represent more than 80% of PIs; for economic impact, the 1E score is shown in 58 PIs, and for organizational impact, 1O score was considered in about 80% of the PIs conducted. Figure 2 illustrates the use of the CLEO tool based on the PIs conducted in the study. A quantitative schematic representation of the use of the CLEO tool based on the PIs conducted in the study can be found in Supplementary Table S3.

More specifically, in terms of clinical impact, the PIs had a positive relevance for pathologies such as lung cancer, breast cancer, colorectal cancer and prostate cancer, with dexamethasone, filgrastim and ondansetron as the main interventional active substances. Supplementary Figure S1 shows the baseline pathologies that had a major and moderate clinical impact with PIs and Supplementary Figure S2 shows the active substances that received PIs and had a minor clinical impact on patients’ health.

**Figure 3:**
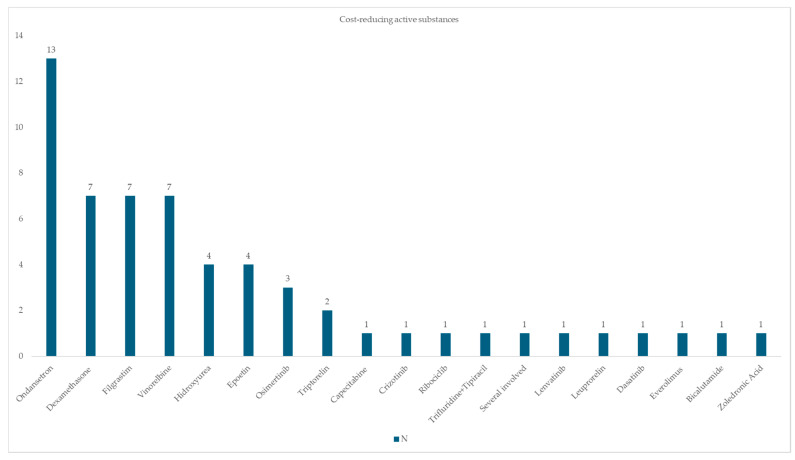
Active substances whose PIs reduced hospital treatment costs. Note. Several involved (apalutamide, ciproterone, bicalutamide, leuprorelin).

In terms of the direct treatment costs, PIs involving active substances such as ondansetron, dexamethasone, filgrastim and vinorelbine, commonly administered for lung, breast and prostate cancer, resulted in reduced treatment costs and overall savings for the hospital (Figure 3 and Figure S3).

**Figure 4:**
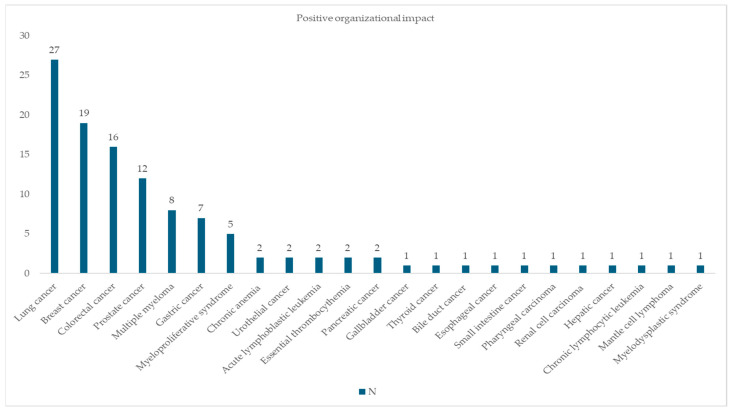
Pathologies impacted by positive organizational impact.

Lung, breast and colorectal cancer also showed a positive organizational impact, particularly for ondansetron, dexamethasone and filgrastim. Figure 4 shows the results for positive organizational impact (score 1O). This positive contribution involved reducing the time spent on correcting prescriptions, improving clinical workflows, and improving interdisciplinary communication between pharmacists and prescribers.

The most common DRPs identified in the study were also categorized according to the three dimensions of the CLEO tool. Regarding clinical impact, for DRPs such as incorrect frequency/time of administration, drug–drug interaction and incorrect dose, scores 1C, 2C and 3C represented the highest proportion compared to score 0C. These results highlight the effectiveness of PIs in addressing these specific DRPs. A similar trend was observed for organization impact, with results concentrated in score 1O. In contrast, for economic impact in these specific DRPs, score 1E was not the most frequently reported, with scores minus 1E and 0E being more prevalent. These results can be found in Table S3.

## 4. Discussion

This study evaluated the predicted clinical, economic, and organizational impact of PIs in a real-world oncology setting using the CLEO multidimensional tool. Our results suggest that PIs frequently addressed DRPs such as incorrect administration frequency, drug–drug interactions, and dosing errors, which aligns with the DRP profiles reported in similar studies conducted in oncology pharmacy practice [10,23]. Corrective actions, primarily prescription modifications, were predicted to enhance medication safety and optimize treatment regimens by reducing the likelihood of therapeutic failure and avoidable harm. These observations align with previous literature emphasizing the pharmacist’s contribution to minimizing medication-related risks in oncology care [5,24].

When comparing our results to studies applying the CLEO tool, similar trends emerge. For example, a Swiss study conducted in an internal medicine setting reported that 97.8% of DRPs were followed by PIs with an expected clinical benefit, while the economic impact was mixed, with both increases and reductions in immediate therapy costs observed [6]. Likewise, our data demonstrated a substantial clinical benefit, with over 80% of interventions rated between 1C and 3C on the CLEO scale, and a substantial proportion showed predicted economic and organizational contributions. The proportion of interventions with economic and organizational impact in our study (40.3% and 79.9%, respectively) is comparable to reports from other European hospitals using structured impact assessment frameworks [4,6].

Importantly, our findings are also aligned with those of Clarenne et al. [8], who conducted a five-year observational study on oncology wards and demonstrated that CLEO-based documentation not only quantifies the multidimensional impact of PIs but also enables benchmarking across institutions. Their analysis highlighted that the systematic evaluation of PIs contributes to the recognition of the pharmacist’s clinical value and supports healthcare decision-making. Similarly, our experience suggests that CLEO provides a transparent framework that can support quality improvement and resource allocation in oncology practice.

In our cohort, most DRPs were linked to incorrect dosing frequency or administration timing, and their resolution was predicted to improve medication safety by avoiding both underdosing (risking therapeutic failure) and overdosing (risking unnecessary toxicity). Other frequent categories included potential drug–drug interactions, inappropriate duplications of supportive care agents (e.g., ondansetron), and dosing inconsistencies, all of which were addressed through prescription modifications or clarifications with the medical team. Beyond clinical outcomes, several interventions may also have had economic consequences—mainly cost avoidance—by preventing medication waste and avoiding potential adverse events. Organizationally, interventions contributed to workflow efficiency, reduced time expenditure for healthcare professionals, and enhanced overall medication-use safety within the oncology department.

The predominance of DRPs related to dosing frequency deserves special consideration. This trend likely reflects the inherent complexity of oncology regimens, which frequently involve multiple agents with strict administration schedules, sometimes requiring modifications in response to adverse effects or patient-specific factors. Corticosteroids such as dexamethasone exemplify this dynamic: their administration may be adapted according to tolerance or toxicity, increasing the risk of discrepancies. Moreover, the complexity of treatment schedules can hinder patient comprehension and increase the potential for prescribing or administration errors.

Regarding the drugs most frequently implicated—dexamethasone, filgrastim, and ondansetron—this pattern is attributable to their central role in supportive care and treatment of the most prevalent cancers in our cohort (lung, breast, prostate, colorectal cancer, and multiple myeloma). Dexamethasone, used as an anti-inflammatory, antiemetic adjuvant, and for edema control, is subject to variable dosing and tapering regimens, which enhances the likelihood of inconsistencies. Filgrastim administration requires precise timing relative to chemotherapy cycles, with frequent adjustments to prevent underuse or overuse. Similarly, ondansetron is prescribed both prophylactically and as rescue therapy for chemotherapy-induced nausea and vomiting, creating scenarios for duplication or frequency-related discrepancies.

Although a formal cost-effectiveness analysis was not conducted, many interventions in our study prevented medication errors—such as incorrect dosing, administration frequency, therapy duplication, and drug–drug interactions—that are well-documented contributors to increased healthcare costs [25]. Thus, our results suggest a potential for pharmacist interventions to optimize resource utilization by reducing the risk of costly adverse events. However, claims of cost-effectiveness cannot be made, as this would require formal modeling of pharmacist time, hospitalization rates, re-admissions, and other health service costs, which were beyond the scope of this study.

An important strength of this work is its demonstration of CLEO’s applicability in a Portuguese oncology department, despite the absence of a formally validated Portuguese version during the study period. The ongoing cultural adaptation and validation of CLEO_pt will enable broader implementation and comparability of PI assessments across Portuguese-speaking healthcare systems.

Several limitations must be acknowledged. First, the retrospective single-center design restricts generalizability, and the relatively small sample size prevents extrapolation to broader populations. Second, all data were reviewed exclusively by pharmacists, raising the possibility of professional bias; no independent validation by other healthcare professionals (e.g., physicians or nurses) was performed. It should also be clarified that the pharmacists reviewed interventions carried out by their peers within the same institution, which may further introduce bias. Third, no control arm was included, and therefore, the observed benefits should be interpreted as predicted or expected, not demonstrated causal outcomes. Finally, long-term patient outcomes and patient-reported measures were not evaluated, and differences in DRP severity by patient characteristics were beyond the scope of this analysis.

Despite these limitations, our study illustrates that CLEO can be applied in a Portuguese oncology department to systematically assess pharmacist interventions. The ongoing cultural adaptation and validation of CLEO_pt will further enable broader implementation and comparability of PI assessments across Portuguese-speaking healthcare systems.

Regarding the implications for practice, our observations support the potential of pharmacist interventions to strengthen medication safety in oncology. The structured use of the CLEO tool provides a standardized approach to documenting and communicating the predicted value of PIs, supporting quality improvement, benchmarking, and informed discussions with healthcare teams and administrators. For patients, this structured approach may translate into safer and more effective therapies, a reduced risk of medication errors, and overall improvements in the quality of care.

In summary, while the evidence presented here is limited by its retrospective single-center design and absence of a control arm, the findings suggest that pharmacist interventions have the potential to deliver multidimensional benefits in oncology care. The CLEO tool emerges as a practical framework for systematically evaluating and communicating these predicted impacts, supporting collaborative decision-making and fostering a culture of medication safety in cancer treatment.

## 5. Conclusions

This study demonstrates that PIs, assessed using the CLEO multidimensional tool, generate clinical, economic, and organizational benefits in oncology care. These findings confirm the feasibility of applying CLEO in a Portuguese hospital setting and highlight its value as a structured approach for documenting and assessing pharmacist-led interventions. By promoting standardized evaluation, CLEO can strengthen the integration of pharmacists within multidisciplinary teams and foster a culture of medication safety. However, the single-center retrospective design and absence of long-term outcomes limit generalizability. Future research should include multicenter prospective studies, assess patient-reported outcomes, and incorporate cost-effectiveness analyses to further validate the impact of PIs in oncology practice.

## Figures and Tables

**Table 1:** Baseline demographic and clinical characteristics of patients who received PIs.

Variable Patients	N	%
Sex		
Male	58	40.3
Female	86	59.7
Age group (Years)		
<18 years	0	0
18–64 years	47	32.6
65–85 years	93	64.6
>85 years	4	2.8
Base Pathology		
Acute lymphoblastic leukemia	5	3.5
Acute myeloid leukemia	1	0.7
Bile duct cancer	1	0.7
Breast cancer	29	20.1
Chronic anemia	2	1.4
Chronic lymphocytic leukemia	1	0.7
Colorectal cancer	16	11.1
Esophageal cancer	2	1.4
Essential thrombocythemia	2	1.4
Gallbladder cancer	1	0.7
Gastric cancer	7	4.9
Hepatic cancer	1	0.7
Lung cancer	29	20.1
Mantle cell lymphoma	1	0.7
Monoclonal gammopathy	1	0.7
Multiple myeloma	13	9.0
Myelodysplastic Syndrome	3	2.1
Myeloproliferative Syndrome	5	3.5
Pancreatic cancer	2	1.4
Pharyngeal carcinoma	2	1.4
Prostate Cancer	15	10.4
Renal Cell Carcinoma	1	0.7
Small intestine cancer	1	0.7
Tyroid cancer	1	0.7
Urothelial cancer	2	1.4
Total	144	100

**Table 2:** Active substances involved in the PIs.

Pharmacotherapeutic Group	Active Substance	N	%
Serotonin (5-HT3) Antagonists	Ondansetron	28	19.4
Immunostimulants	Filgrastim	20	13.9
Antianemic preparations	Epoetin	5	3.5
Corticosteroids for systemic use	Dexamethasone	18	12.5
Tyrosine Kinase inhibitors	Cabozantinib	1	0.7
	Crizotinib	1	0.7
	Dabrafenib	1	0.7
	Dasatinib	4	2.8
	Imatinib	2	1.4
	Ibrutinib	1	0.7
	Lenvatinib	3	2.1
	Osimertinib	3	2.1
	Ribociclib	2	1.4
Antineoplastic agents, cytotoxic	Chlorambucil	1	0.7
	Docetaxel + Cyclophosphamide	1	0.7
	Hidroxyurea	5	3.5
	Vinorelbine	8	5.6
Antimetabolite	Capecitabine	7	4.9
	Trifluridine + Tipiracil	2	1.4
Antiandrogens	Bicalutamide	4	2.8
	Several involved (apalutamide, ciproterone, bicalutamide, leuprorelin) ^1^	1	0.7
	Enzalutamide	2	1.4
Immunosuppressants	Lenalidomide	2	1.4
	Talidomide	4	2.8
Gonadotropin-Releasing Hormone analogs	Triptorelin	2	1.4
	Leuprorelin	3	2.1
Anti-estrogens	Tamoxifen	5	3.5
Bisphosphonates	Zoledronic acid	2	1.4
Antidiabetics	Various oral antidiabetics	1	0.7
Antidyslipidemic	Ezetimibe	1	0.7
Immunostimulating agent	Interferon alfa 2a	1	0.7
B-cell lymphoma (BCL)-2 inhibitor	Venetoclax	1	0.7
Aromatase Inhibitor	Letrozol	1	0.7
Mammalian target of rapamycin (mTOR) kinase inhibitors	Everolimus	1	0.7
Total		144	100

^1^ These active substances were prescribed in two medical prescriptions to the same patient.

## Data Availability

The original contributions presented in this study are included in the Original Article/Supplementary Materials. Further inquiries can be directed at the corresponding author(s).

## Supplementary Materials

The following supporting information can be downloaded at: https://www.mdpi.com/article/10.3390/pharmacy13050143/s1, Figure S1: Baseline pathologies with major and moderate clinical impact; Figure S2: Active substances that received PIs and had a minor clinical impact on patient’s health; Figure S3: Pathologies whose PIs represented a reduction in the cost of treatment for the hospital; Table S1: Drug-related problems considered in the study; Table S2: Actions taken during PIs; Table S3: CLEO impact assessment by PIs conducted in the study; Table S4: CLEO impact assessment based on the most common DRPs identified in the study.
