# Supplementary material for: Impact of Pharmacist Interventions in a Portuguese Hospital: A Study Using the CLEO Multidimensional Tool
Source: Pharmacy (Basel). 2025 Oct 5;13(5):143. doi: 10.3390/pharmacy13050143 (PMC12567203; doi:10.3390/pharmacy13050143)
Supplement: Supplementary file 1 [file pharmacy-13-00143-s001.zip › pharmacy-3752495-supplementary.pdf]

Supplementary material

Table S1. Drug-related problems considered in the study.

	Drug-related problem
1	Medication not indicated / not appropriate for the diagnosis
2	History of allergy or similar adverse reaction
3	Inappropriate medication for the patient due to age, clinical situation, etc.
4	Contraindicated medication
5	Drug-drug interaction
6	Drug-food interaction
7	Duplication of therapy
8	Unnecessary medicines
9	Incorrect dose
10	Incorrect pharmaceutical form
11	Incorrect route of administration
12	Lack of specific administration instructions
13	Incorrect frequency/time of administration
14	Lack of drug identification
15	Incorrect/unspecified duration of treatment
16	Medication omission
17	Incorrect patient
18	Inconsistent information (e.g. prescribed dose different from the information on the drug's observation line)
19	Treatment discontinued due to toxicity
20	Treatment discontinued due to lack of efficacy
21	Other reasons

Note. To support the understanding of the classification applied, representative examples of clinical cases corresponding to some of the eight DRP categories identified in our study are provided below:

Wrong frequency or time of administration – *e.g., a 72-year-old patient was prescribed ondansetron and dexamethasone to start on 5 July, although chemotherapy was scheduled for 10 July. The treatment initiation was therefore adjusted to align with the chemotherapy start date, avoiding unnecessary early administrations.*

Drug–drug interaction (manifest or potential) – *e.g., a 58-year-old patient receiving amiodarone was prescribed leuprorelin. Due to a major drug–drug interaction, administration of leuprorelin was postponed pending cardiology evaluation, with the drug kept available for nursing staff until medical clearance. This intervention avoided potential cardiovascular complications.*

Incorrect dose (too high or too low) – *e.g., a 77-year-old patient was prescribed lenvatinib 20 mg, while the clinical record indicated 24 mg. After confirmation with the oncologist, the correct dose of 20 mg was maintained. This intervention ensured accurate dosing and avoided potential medication error.*

Medication not indicated/not appropriate for diagnosis – *e.g., a 56-year-old patient was prescribed peginterferon beta-1a, although the clinical record indicated ongoing therapy with pegylated interferon alfa-2a at a reduced dose. After clarification with the physician, the prescription error was corrected, and the patient continued interferon alfa-2a at the adjusted dose. This intervention prevented inappropriate drug substitution and ensured therapeutic continuity.*

Therapy duplication – *e.g., during medication reconciliation, a discrepancy was identified: the patient was taking four antidiabetic drugs (metformin, dapagliflozin, gliclazide, and sitagliptin/metformin), which was not consistent with the clinical records. The physician was informed, and the case was referred to the primary care provider for therapeutic reassessment. This intervention ensured accurate medication documentation and highlighted potential overprescription.*

Table S2. Actions taken during PIs.

	N	%
**Action taken**		
Correct prescription	68	47,2
Maintain prescription	43	29,9
Not applicable	33	22,9
**Total**	**144**	**100**

Table S3. CLEO impact assessment by PIs conducted in the study.

	N	%
**Clinical impact**		
**Score**		
Minus 1C	0	0
0C	28	19,4
1C	45	31,3
2C	46	31,9
3C	25	17,4
4C	0	0
ND	0	0
**Economic impact**		
**Score**		
Minus 1E	11	7,6
0E	75	52,1
1E	58	40,3
ND	0	0
**Organizational impact**		
**Score**		
Minus 1O	8	5,6
0O	21	14,6
1O	115	79,9
ND	0	0
**Total**	**144**	**100**

ND – Not defined.

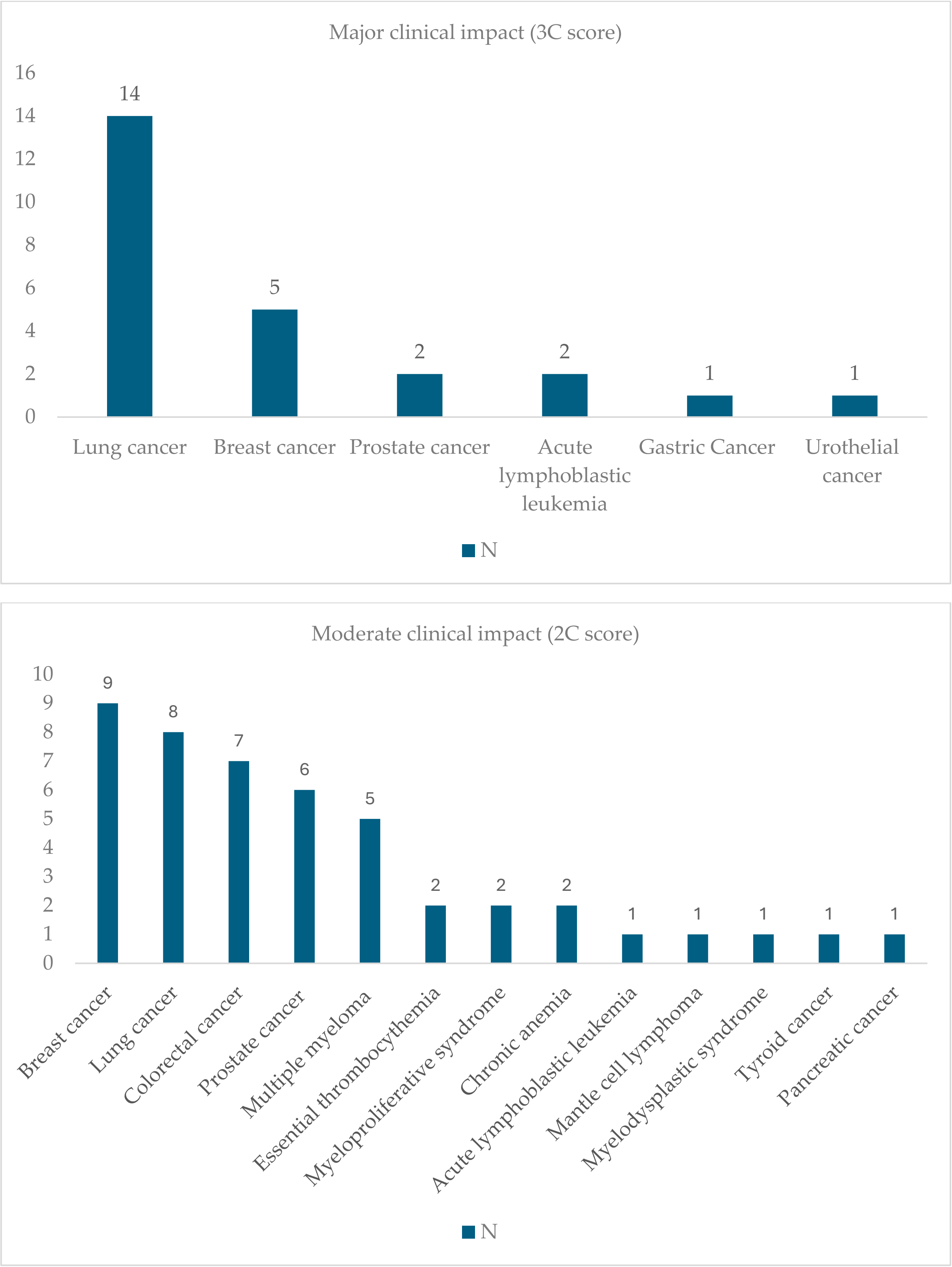

Figure S1. Baseline pathologies with major and moderate clinical impact.

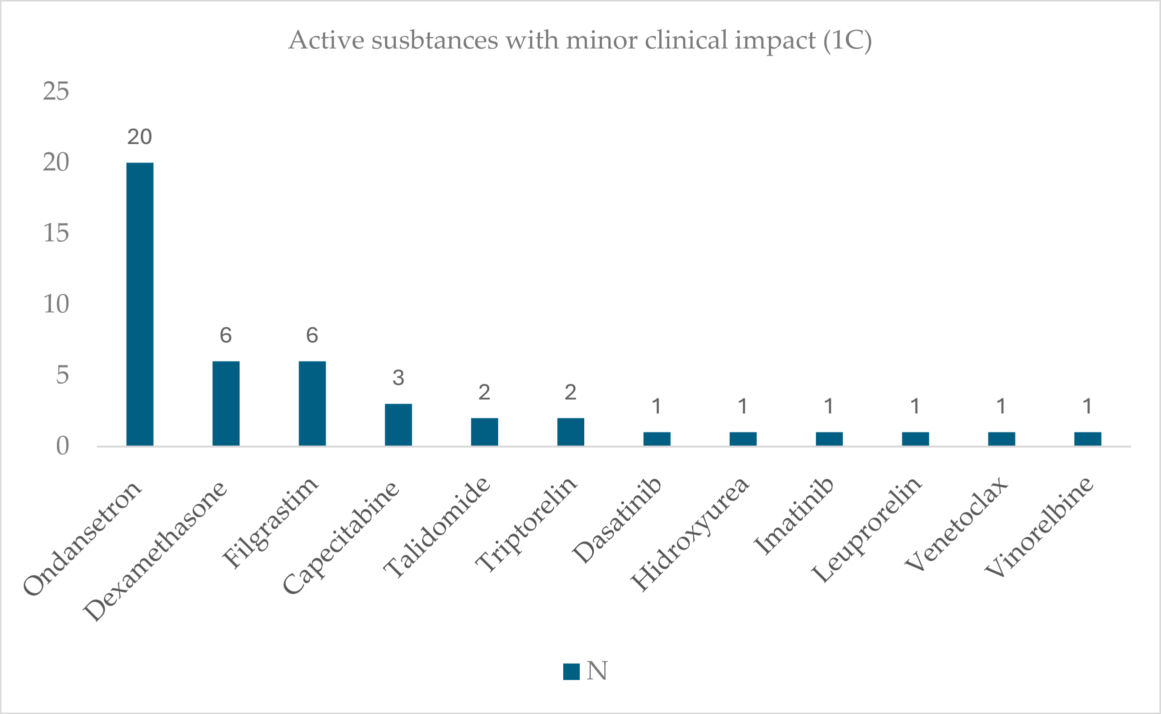

Figure S2. Active substances that received PIs and had a minor clinical impact on patient's health.

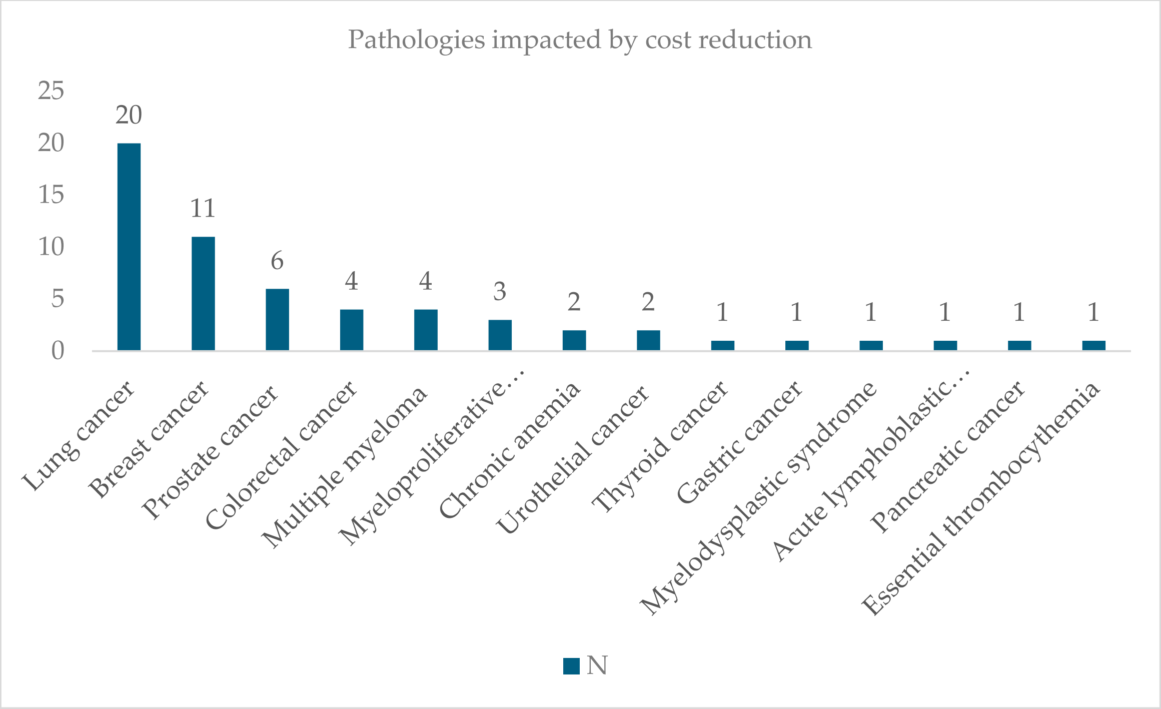

Figure S3. Diseases in which PIs contributed to lowering treatment costs for the hospital.

Table S4. CLEO impact assessment based on the most common DRPs identified in the study.

		N	% (of the total 144 interventions)
**Drug-related problem**			
**Incorrect frequency/time of administration**			
Clinical impact	0C	12	8,3
	1C	33	22,9
	2C	20	13,9
	3C	18	12,5
Economic impact	Minus 1E	5	3,5
	0E	35	24,3
	1E	43	29,9
Organizational impact	0O	9	6,3
	1O	74	51,4
**Drug-drug interaction**			
Clinical impact	0C	10	6,9
	1C	6	4,2
	2C	13	9,0
	3C	3	2,1
Economic impact	Minus 1E	1	0,7
	0E	28	19,4
	1E	3	2,1
Organizational impact	Minus 1O	6	4,2
	0O	8	5,6
	1O	18	12,5
**Incorrect dose**			
Clinical impact	0C	3	2,1
	1C	2	1,4
	2C	8	5,6
	3C	2	1,4
Economic impact	Minus 1E	3	2,1
	0E	4	2,8
	1E	8	5,6
Organizational impact	0O	4	2,8
	1O	11	7,6
